# El Dourine

**Published:** 1891-04

**Authors:** W. H. Ridge


### EL DOURINE.*

By W. H. Ridge, V. M. D.

Case.—Gray gelding, about 15.3 hands high ; weight, about 1,050 pounds ; six years old. Examined him, September 26, 1890. Found that the owner had bought him at a bazaar in Philadelphia a few days before. I saw that he had a peculiar attitude ; penis out part-way, which owner said occured a great part of the time ; knuckled badly. Was extremely sensitive over the loins, almost lying down when pressed upon. Inside of legs were badly soiled with a discharge that came from the urethra. The mm of mouth was denuded in large patches, leaving angry ulcers, with vesicles covering the unbroken parts. The collection of masticated food had fermented, making foul smell to breath. Nostrils clear, rosy. In the centre of forehead I found a cicatrix which was depigmented. Around each upper eye-lid there was a scab which was easily detatched, leaving a white scar. The mucous membrane was normal. The sub-maxillary lymphatic glands were enlarged, but not nodulated nor attached high up as in glanders. On taking the temperature, which was 100½° F., I found large patches around the anus which were depigmented, leaving me with the idea that there had been ulcers there and they had cicatrized, as the one in forehead and eyes. No swellings as in melanosis. I found ulcers on penis and sheath, many patches were dry, scaling off, other places were discharging, the end of urethra swollen and discharging a sticky fluid which had soiled the inside of the thighs and legs. The owner said his appetite was ravenous. Noticed that he changed positions a great deal ; on motion, went something as if sore, but on closer observation, could see that it resembled loss of co ordination (locomotor ataxia). Owner said there were parts that seemed to itch him, as he would nib and bite the skin. Could see no signs of recent castration.

My diagnosis at that time was variola, although we had a low temperature for an animal in this condition. I next saw him on October 3d, when I made a diagnosis of Dourine and requested a consultation at the Veterinary Department, Univ. of

* Case reported at the meeting of the Pennsylvania State Veterinary Medical Association, March 3rd.

Pa., to confirm my diagnosis—condition much the same except there are signs of paralysis of the hind parts, so much so that the owner said he was afraid to try to drive him, although his will, energy and appetite seemed all right—prescribed Pot. Iod. ℥ i ; Fowlers Sol. ℥ iv ; teaspoonful, t. i. d. Oct. 5th saw him again owner would not take him away. Temp. 100 pulse 45 Resp. 14, looks bright much the same, only find little elevations much like surfeit, on the skin. I ordered the mouth and sheath washed with borax solution.

Oct. 14. Found that many of the layity have made a positive diagnosis. Some persons having syphilis had urinated on some sore, giving the animal syphilis, I mention this point, as I find there are some who practice Veterinary Medicine that think such possible. I induced the owner to ship him to the Veterinary Dept. which he did to-day—one point that struck me was the change of the depigmented spots around the anus, without any ulcers forming, as some were nearly an inch from the primary spots.

Now the weather up to this time has been foggy, damp and warm, after sending we had a change to freezing, and clear, the animal appeared in the clinics on the 15th inst., when Dr. Zuill confirmed my diagnosis of El Dourine and put him on hygienic treatment without any internal remedies. Owner took him away, on Oct. 20 and came to me for some of the same medicine that Dr. Zuill had given the animal, as he was much better than any time before. But on his way home the owner fell in with someone who told him about the virtues of "life everlasting." I prescribed Quinine grs. ii, Ferri Sulph ʒ ss twice a· day, with the understanding that he could give all the "life everlasting" he wished. I found that they had inoculated an old horse at the University Veterinary Hospital by means of a sponge, but with no result.

Oct. 23, I found the mouth nearly all healed leaving white spots, the sheath not so much soiled but nervous symptoms much the same.

I prescribed Quinine Sulph. and Ferri Sulph. in powders. Also gave, Fowlers Sol and Tr. Nux. Vom. equal parts, teaspoonful night and morning. Nov. 17. I drove the horse 7 miles, I could scarcely see any thing the matter, no ulcers, no discharge no ataxia, coat looked bright and seemed in good health. Owner seemed to attribute it all to "life everlasting," so I stopped my treatment. Dec. 7, he sent for me, the mouth much the same as at first, urethra discharging, there is a sore over loins about the size of my hand. Prescribed Pot. Iod. and Fowlers Sol. when he began to improve, but yet to-day March 3d there is enough I think for any one to make a diagnosis.

